# Mitochondrial Dysfunction and Infection Generate Immunity–Fecundity Tradeoffs in *Drosophila*

**DOI:** 10.1093/icb/icy078

**Published:** 2018-07-24

**Authors:** Justin L Buchanan, Colin D Meiklejohn, Kristi L Montooth

**Affiliations:** School of Biological Sciences, University of Nebraska–Lincoln, 1104 T St, Lincoln, NE 68588-0118, USA

## Abstract

Physiological responses to short-term environmental stressors, such as infection, can have long-term consequences for fitness, particularly if the responses are inappropriate or nutrient resources are limited. Genetic variation affecting energy acquisition, storage, and usage can limit cellular energy availability and may influence resource-allocation tradeoffs even when environmental nutrients are plentiful. Here, we utilized *Drosophila* mitochondrial–nuclear genotypes to test whether disrupted mitochondrial function interferes with nutrient-sensing pathways, and whether this disruption has consequences for tradeoffs between immunity and fecundity. We found that an energetically-compromised genotype was relatively resistant to rapamycin—a drug that targets nutrient-sensing pathways and mimics resource limitation. Dietary resource limitation decreased survival of energetically-compromised flies. Furthermore, survival of infection with a natural pathogen was decreased in this genotype, and females of this genotype experienced immunity–fecundity tradeoffs that were not evident in genotypic controls with normal energy metabolism. Together, these results suggest that this genotype may have little excess energetic capacity and fewer cellular nutrients, even when environmental nutrients are not limiting. Genetic variation in energy metabolism may therefore act to limit the resources available for allocation to life-history traits in ways that generate tradeoffs even when environmental resources are not limiting.

## Introduction

The energy available to heterotrophic organisms is often determined by nutrients in the environment, and the dynamic allocation of these resources within the lifespan of an individual impacts life-history tradeoffs between organismal maintenance and reproduction. Nutritional stress may be caused by the lack of a single nutrient (Bergland et al. 2008; Jensen et al. 2015), improper nutrient ratios (Skorupa et al. 2008), or reduced overall food availability leading to a decrease in overall calorie consumption. Energetic costs associated with infection are predicted to have a significant impact on survivorship and future reproduction via the allocation of limited resources between reproduction and immunity (Lochmiller and Deerenberg 2000; Harshman and Zera 2007; Schwenke et al. 2016). Energetic costs of infection can be associated with the mechanisms of pathogen resistance (e.g., constitutive and induced immune responses) and tolerance (Rauw 2012), reduced nutrient uptake during infection (Bonfini et al. 2016), or resource consumption by pathogens (Cressler et al. 2014; Kurze et al. 2016).

Despite the prediction that fighting infection will generate a tradeoff with future reproduction, the relationship between infection and reproduction is complex. Under some conditions, adult infection decreases fecundity and the expression of reproduction genes (Short and Lazzaro 2013). However, constitutive immune expression does not always generate life-history tradeoffs (Fellous and Lazzaro 2011), and infection can even increase fecundity (Adamo 1999) and offspring quality (Stahlschmidt et al. 2013; Reavey et al. 2015). Increased reproduction post-infection may occur via parasite manipulation (e.g., Weeks and Stouthamer 2004) or if hosts switch resources toward short-term investment in reproduction (Cressler et al. 2015), a strategy known as terminal investment (Clutton-Brock 1984; Bonneaud et al. 2004). Understanding how host energy metabolism impacts resource allocation and immune function, and the consequences for life-history tradeoffs remain an important area of research, with implications for the field of ecological immunology (Sheldon and Verhulst 1996; Brock et al. 2014).

**Fig. 1:**
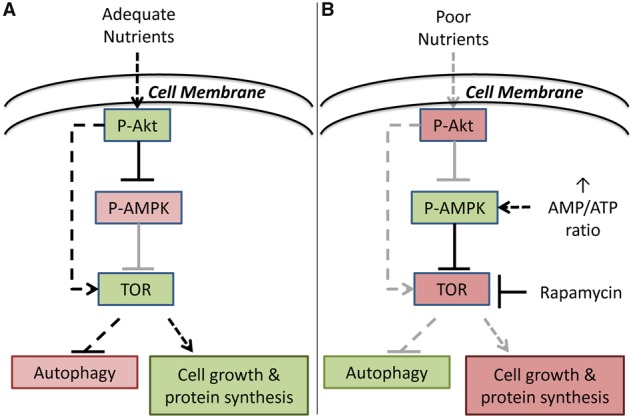
The target of rapamycin (TOR) protein integrates nutrient responses to regulate growth. (**A**) In the presence of adequate nutrients, TOR is active, which represses recycling of cellular components via autophagy and promotes growth. (**B**) When nutrients are sensed as being limited either via insulin signaling, an increased AMP/ATP ratio, or artificially by exposure to the drug rapamycin, TOR is repressed which promotes autophagy and inhibits growth.

Investigating how genetic variation in host metabolism impacts immune function and interacts with diet to influence life-history outcomes during periods of environmental stress (e.g., infection) is critical for understanding the evolution of immunity–fecundity tradeoffs. Genetic variation affecting energy metabolism may limit the availability of cellular energy (e.g., Adenosine triphosphate [ATP]) and influence resource-allocation tradeoffs even when environmental nutrients are not limiting. Thus, the extent to which environmental nutrients are limiting is expected to vary among individuals. One regulatory mechanism that integrates information from external (e.g., food availability) and internal (e.g., ATP) inputs is the target of rapamycin (TOR) signaling pathway (Oldham and Hafen 2003). When external and internal nutrient levels are sufficient, TOR upregulates downstream genes to promote protein synthesis and growth. Conversely, poor nutrient levels or treatment with the drug rapamycin decreases protein production and increases recycling of cellular components via autophagy, slowing growth (Zheng et al. 1995; Hahn-Windgassen et al. 2005; Fig. 1). Consistent with these effects, rapamycin delays development, decreases fecundity, and increases lifespan in the fruit fly *Drosophila melanogaster* (Bjedov et al. 2010).

To investigate how genetic variation in energy metabolism and, specifically, in mitochondrial function affects immune function and immunity–fecundity tradeoffs, we utilized a mitochondrial–nuclear (mito–nuclear) genotype of *Drosophila* that compromises mitochondrial oxidative phosphorylation (OXPHOS). Compromised OXPHOS in this genotype is caused by an incompatible interaction between a single nucleotide polymorphism in the mitochondrial-encoded mt-tRNA^Tyr^ and an amino acid polymorphism in the nuclear-encoded mt-tyrosyl-tRNA synthetase that aminoacylates this mt-tRNA (Meiklejohn et al. 2013). Together, these mutations disrupt larval metabolism, delay development, and decrease female fecundity, indicative of inefficient energy metabolism (Hoekstra et al. 2013, 2018; Meiklejohn et al. 2013). Here we measured life-history traits in mito–nuclear genotypes under nutrient- and pathogen-stress conditions to test whether genetic variation that compromises energy metabolism can limit available cellular resources and generate tradeoffs between immunity and fecundity.

## Methods

### *Drosophila* genotypes and rearing conditions

We employed six mito–nuclear genotypes that combine mtDNAs from *Drosophilasimulans*—(*simw^501^*) and (*sm21*)—and *D. melanogaster* (*ore*) with two wild-type *D. melanogaster* nuclear genomes—*OreR* and *Aut* (Montooth et al. 2010). Of these six genotypes, only the (*simw^501^*); *OreR* mito–nuclear combination generates an incompatible interaction that decreases OXPHOS; the other five genotypes serve as wild-type controls. All genotypes were maintained at 25°C with a 12 h:12 h, light:dark cycle. Three non-isocaloric food types were used in experiments: our standard laboratory food, which is a high-yeast diet (0.88% agar, 8.33% Torula yeast, 10% Cornmeal, 0.33% Tegosept W/V and 4.66% Molasses, 1.66% 95% ethanol, and 0.66% propionic acid V/V dH_2_O), a low-yeast diet (our standard food with 0.5% Torula Yeast W/V), and a medium-mixed diet (0.93% agar, 2.94% SAF Yeast, 6.12% Cornmeal, 12.94% sugar, 0.28% Tegosept W/V and 1.08% 95% ethanol, and 0.71% propionic acid V/V dH_2_O).

### Rapamycin and diet effects on development

To test whether the energetically-compromised (*simw^501^*); *OreR* genotype has disrupted nutrient-sensing, we developed all six genotypes from egg to adult on the medium-mixed diet containing three concentration of rapamycin concentrations (0, 2, and 10 µM). Fifty females and 30 males of each genotype were mated for 24 h and placed onto grape-agar plates (50 g bacto-agar, 30 mL tegosept in 10% ethanol, 500 mL grape juice, 1500 mL distilled H_2_O) for collecting cohorts of eggs every 24 h. A total of five replicate vials of 75 eggs per genotype and rapamycin concentration were monitored twice a day to measure the development time of each individual and the number of males and females that eclosed as a measure of sex-specific survival. This assumed a 50:50 sex ratio in the eggs or larvae (see below) placed in each vial.

In order to examine additional rapamycin concentrations, genotypes with the (*sm21*) mtDNA—which did not behave differently from the (*ore*) control mtDNA in the initial experiment—were not included in a second experiment. In this experiment, four genotypes were reared on the high-yeast diet for many generations before being reared on food containing 0, 5, 10, or 15 µM rapamycin. Males and females of each genotype were mated, and females were allowed to lay eggs for 12 h on grape-agar plates. Fifty first-instar larvae of each genotype were collected 24 h later. Seven to eight replicate vials of each genotype at each rapamycin concentration were measured for development time and survival as described above.

In order to test the prediction that control genotypes exposed to a low-yeast diet would show a decreased responsiveness to rapamycin, similar to (*simw^501^*); *OreR* (see the “Results” section), we developed all six mito–nuclear genotypes from larvae to adult on either a high-yeast or low-yeast diet, supplemented with 0, 5, or 10 µM rapamycin. Males and females of each genotype were mated, and females were allowed to lay eggs for 4h on high- or low-yeast plates. One hundred first-instar larvae of each genotype were collected 30 h after the egg lay. Five replicate vials of each genotype, yeast, and rapamycin combination were measured for development time and survival as described above.

### Bacterial infection and female fecundity

To test whether compromised energy metabolism decreases the ability to survive bacterial–pathogen infection, we infected virgin 1-day old adults of all six mito–nuclear genotypes with the natural pathogen *Providencia rettgeri* (Juneja and Lazzaro 2009; Short and Lazzaro 2013). Individuals were either sham infected with 1× PBS or infected with *P. rettgeri* in 1× PBS at a concentration of 1.0 OD (∼5000 bacterial cells) using a 0.1 mm needle (TedPella 13561-50) (Khalil et al. 2015). The infection protocol results in moderate lethality: 40–80% of adults survive depending on the infection method and condition of flies, with infection stabilizing by day 4 (Sackton et al. 2010; Howick and Lazzaro 2014; Duneau et al. 2017a). Flies were then placed in groups of 30 males or females on standard food and survivors were counted twice daily for 10 days. Five replicate groups of each genotype, sex, and infection treatment (sham vs. pathogen) combination were measured for survival. In a parallel infection setup, fecundity was measured using 15–20 females of each genotype–treatment combination that had survived to 5 days post infection. These females were mated with wild-type males that were genetically distinct from the focal genotypes. Mated females were allowed to lay eggs for 72 h, transferring both males and females to a new vial every 24 h.

### Statistical analyses

Development time to adult eclosion was analyzed using linear mixed-effects models with mtDNA, nuclear genotype, sex, treatment (rapamycin, diet, infection), and their interactions as fixed effects, and replicate vial as a random variable. Rapamycin concentration was treated as an ordered factor. Tukey’s tests were performed with Holm’s sequential Bonferroni correction. The same fixed effects were included in a generalized linear-model analyses of the proportion of flies surviving treatment in each vial. Cox proportional hazard mixed-effects model estimates of hazard ratios associated with infection were obtained using the coxme function in R (Therneau et al. 2003). Fecundity was analyzed using linear models that included the fixed effects of day, genotype, and treatment. Outliers were identified via the Grubbs test and removed. However, analyses with and without outlier data did not produce qualitatively different results. All analyses were carried out in R version 3.4.2 (R Core Team 2017), and statistical tables are provided in Supplementary Tables. Due to the prevalence of main and interaction effects with sex, as well as extensive evidence of sexual dimorphism for life history and physiology in *Drosophila* (Millington and Rideout 2018), we plotted female and male data separately.

## Results

### Individuals with compromised energy metabolism were resistant to rapamycin

The mito–nuclear genotype (*simw^501^*); *OreR* decreases mitochondrial OXPHOS activity with deleterious effects on metabolic rate, development, and female fecundity that are sensitive to energy demand (Hoekstra et al. 2013, 2018; Meiklejohn et al. 2013; Holmbeck et al. 2015; Zhang et al. 2017). Here we tested whether (*simw^501^*); *OreR* flies had altered nutrient sensing due to their predicted low level of cellular energy even when reared on a non-limiting diet. We raised this genotype and genotypic controls that have normal energy metabolism on diets containing rapamycin. This drug represses TOR, an energy-sensing protein downstream of both the insulin receptor and Adenosine monophosphate (AMP)-activated protein kinase (AMPK)—a central regulator of cellular metabolism that responds to the relative abundances of AMP and ATP. Thus, TOR integrates multiple signals of nutrient availability and energetic status to control growth (Fig. 1).

**Fig. 2:**
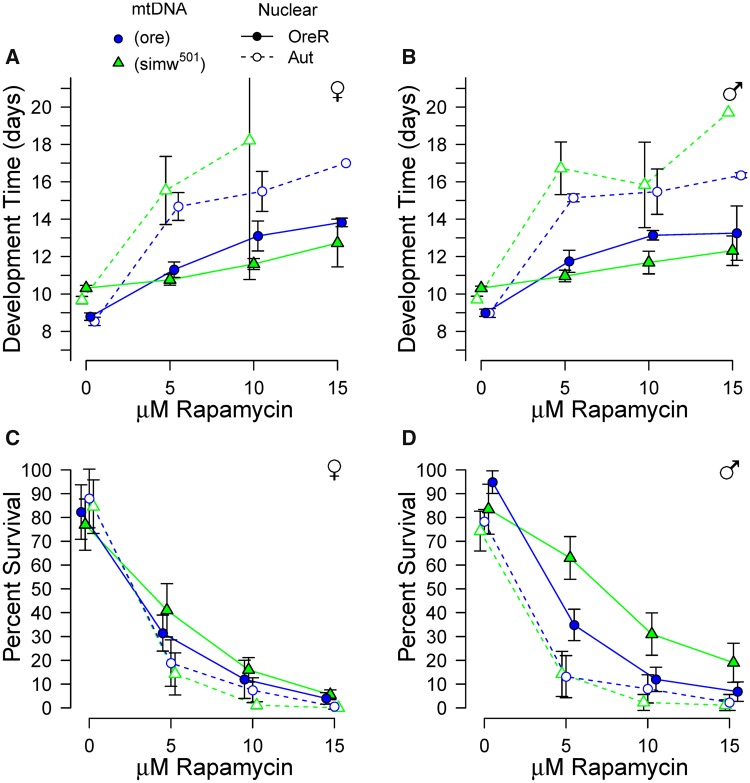
The energetically-compromised genotype (*simw^501^*); *OreR* was relatively resistant to the drug rapamycin. (**A**, **B**) The effect of rapamycin to increase development time was attenuated in (*simw^501^); OreR* relative to control genotypes in both sexes. (**C**, **D**) (*simw^501^*); *OreR* had similar survival to genetic controls in the absence of rapamycin, but had the highest survival in the presence of rapamycin in both sexes. Points are average trait values across seven to eight replicate vials with 95% CI for females (A, C) and males (B, D). Low survivorship of the *Aut* nuclear background accounts for the increase in variance and lack of error bars for development time at high rapamycin concentrations. Statistical results are in Supplementary Table S1 and the main text.

In two independent experiments, we found that rapamycin extended development time of control genotypes in a dose-dependent manner (Fig. 2 and Supplementary Fig. S1), consistent with prior observations in *Drosophila* (Zhang et al. 2000; Wang et al. 2016). However, the energetically-compromised (*simw^501^*); *OreR* genotype was resistant to the effect of rapamycin on development time and survived rapamycin treatment better than control genotypes (Fig. 2 and Supplementary Fig. S1). An interaction between mtDNA genotype, nuclear genotype, and rapamycin concentration significantly affected development time (mtDNA* *×* *nuclear* *×* *rapamycin, *P *<* *0.0001), a pattern that was independent of sex (mtDNA* *×* *nuclear* *×* *rapamycin* *×* *sex, *P *=* *0.14) (Supplementary Table S1). In the experiment on the medium-mixed diet, flies with the *Aut* nuclear genome did not survive at high rapamycin concentrations; in this experiment, an interaction between mtDNA and rapamycin concentration significantly affected development time for individuals with the *OreR* nuclear genome (mtDNA* *×* *rapamycin, *P *<* *0.0001) (Supplementary Fig. S1 and Table S2). In both experiments, the interaction appeared to be driven by an attenuated response of (*simw^501^*); *OreR* development time to rapamycin, relative to the control genotypes (Fig. 2A, B and Supplementary Fig. S1A, B).

In addition to delaying development, rapamycin caused significant dose-dependent mortality in all genotypes (Fig. 2C, D and Supplementary Fig. S1C, D). An interaction between mtDNA genotype, nuclear genotype, and rapamycin concentration significantly affected survival (mtDNA* *×* *nuclear* *×* *rapamycin, *P *<* *0.0003 in both experiments), a pattern that was independent of sex (mtDNA* *×* *nuclear* *×* *rapamycin* *×* *sex, *P *>* *0.39 in both experiments) (Supplementary Tables S1 and S2). Again, this effect was attenuated in (*simw^501^*); *OreR* relative to the control genotypes, with this genotype often having the highest survival in the presence of rapamycin (Fig. 2C, D). This pattern was only observed when first-instar larvae (Fig. 2) rather than embryos (Supplementary Fig. S1) were placed on food containing rapamycin, likely due to high embryonic lethality in this genotype (Zhang et al. 2017). In summary, (*simw^501^*); *OreR* individuals were relatively resistant to the effects of rapamycin on survival to adulthood and development time, suggesting that this genotype may have less responsive TOR signaling as a consequence of a deficient cellular energetic state even when provided a high-nutrient diet.

### The effects of diet and rapamycin were genotype and sex specific

**Fig. 3:**
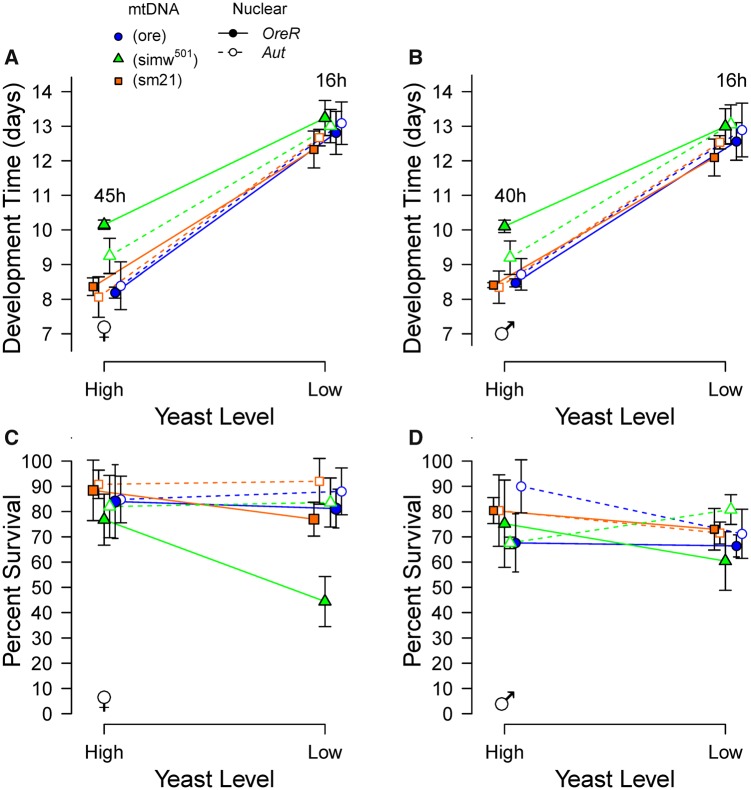
Dietary yeast modified the effects of a mitochondrial–nuclear incompatibility on development time and survival. (**A**, **B**) Decreased dietary yeast delayed development of all genotypes, but the response of (*simw^501^*); *OreR* to dietary yeast was less than that of control genotypes. The differences in average development time in hours between (*simw^501^*); *OreR* and *OreR* nuclear genotypic controls are indicated. (**C**, **D**) (*simw^501^*); *OreR* females, but not males, had decreased larval-to-adult survival relative to control genotypes when developed on a low-yeast diet. Points are average trait values across five replicate vials with 95% CI for females (A, C) and males (B, D). Statistical results are in Supplementary Table S3 and the main text.

Dietary yeast levels affect *Drosophila* development and ovary size (Bergland et al. 2008; Becher et al. 2012). Yeast is an important source of dietary amino acids, and limiting dietary amino acids slow *Drosophila* development, possibly via TOR signaling (Colombani et al. 2003; Oldham and Hafen 2003). We reared mito–nuclear genotypes on both high- and low-yeast diets across a range of rapamycin concentrations to test two hypotheses. We first tested whether (*simw^501^*); *OreR* individuals were relatively resistant to the effects of decreased dietary yeast in the absence of rapamycin treatment. While a low-yeast diet extended development in all genotypes in the absence of rapamycin, the effect was dampened in (*simw^501^*); *OreR* (Fig. 3A, B) (Supplementary Table S3). On a high-yeast diet, the development time of this genotype was delayed by nearly 2* *days, relative to genotypic controls (*P*_females_* *<* *0.05, *P*_males_* *<* *0.01 for all Tukey’s contrasts). However, in a low-yeast environment the developmental time of (*simw^501^*); *OreR* flies was not significantly different from genotypic controls (*P*_females_* *>* *0.38, *P*_males_* *>* *0.44 for all Tukey’s contrasts). This pattern was also observed on the medium-mixed diet that was intermediate in yeast content (Supplementary Fig. S2B and Table S4). The lack of extended development on a low-yeast diet appeared to come at a cost to female survival to adulthood; female (*simw^501^*); *OreR* larval-to-adult survival was significantly reduced to 50% on a low-yeast diet, relative to control genotypes (*P *<* *0.001 for all Tukey’s contrasts) (Fig. 3C), while males had survival that was similar to *OreR* genotypic controls under both diets (*P*_High-yeast_* *>* *0.05, *P*_Low-yeast_* *>* *0.05 for all Tukey’s contrasts) (Fig. 3D and Supplementary Table S3).

**Fig. 4:**
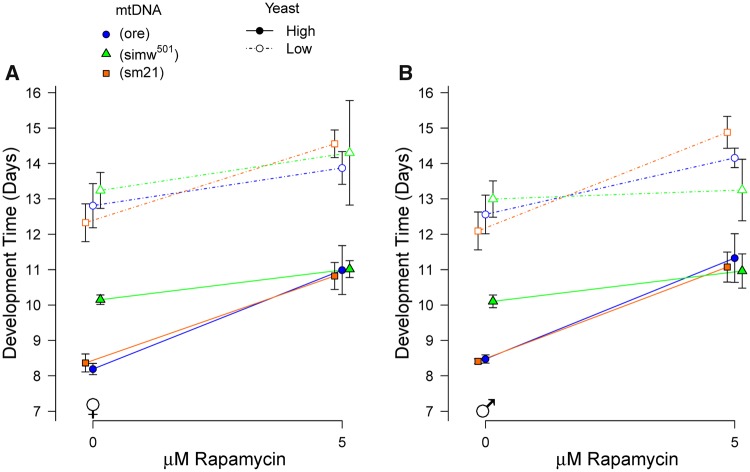
A low-yeast diet attenuated the response of some mitochondrial–nuclear genotypes to rapamycin. Similar to (*simw^501^*); *OreR* on a high-yeast diet, the (*ore*); *OreR* genotype had an attenuated response to rapamycin when fed a low-yeast diet. Points are average trait values across five replicate vials with 95% CI for females (**A**) and males (**B**). Statistical results are in Supplementary Table S5 and the main text.

Second, we aimed to test whether control genotypes developed with decreased dietary nutrients were resistant to rapamycin, in a similar way to (*simw^501^*); *OreR* individuals fed a non-limiting diet. However, flies with the *Aut* nuclear background had very low survival to adulthood when developed on rapamycin, independent of mtDNA genotype. This effect was enhanced on the low-yeast diet, with very few individuals surviving after greatly extended development in the presence of rapamycin. At 10 µM rapamycin on a low-yeast diet, too few flies of all genotypes survived to provide good estimates of development time (Supplementary Fig. S3). However, we were able to use two compatible mito–nuclear genotypes with the *OreR* nuclear background—(*ore*); *OreR* and (*sm21*); *OreR*—to test the prediction that control genotypes fed a low-yeast diet would be less responsive to 5* *µM rapamycin, similar to the (*simw^501^*); *OreR* genotype. Consistent with this prediction, (*ore*); *OreR* flies developed on a low-yeast diet had a dampened response of development time to 5 µM rapamycin, relative to (*ore*); *OreR* flies developed on a high-yeast diet (yeast* *×* *rapamycin, *P *=* *0.007), an effect that was independent of sex (yeast* *×* *rapamycin* *×* *sex, *P *=* *0.11) (Fig. 4 and Supplementary Tables S5 and S6). However, this pattern was not observed in (*sm21*); *OreR* (yeast* *×* *rapamycin, *P *=* *0.85; yeast* *×* *rapamycin* *×* *sex, *P *=* *0.45) (Fig. 4 and Supplementary Tables S5 and S6). Together, our results indicate that nutrient limitation—either in the diet or by mutations affecting energy metabolism—can attenuate delays in larval development due to nutrient-signaling via TOR.

### Energetically-compromised individuals had decreased immune function

**Fig. 5:**
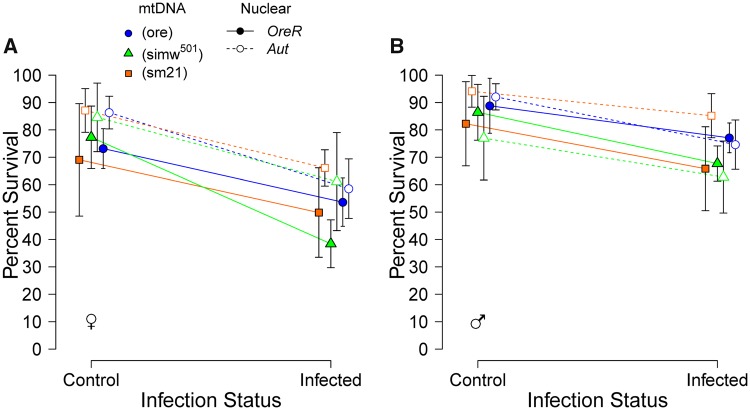
The energetically-compromised genotype (*simw^501^*); *OreR* had decreased survival of infection with the natural pathogen *P. rettgeri*, relative to control genotypes, an effect that was greater in females (**A**) than in males (**B**). Control refers to sham infection. Points are averages across five to six replicate vials with 95% CI. Survival plots are provided in Supplementary Fig. S4. Statistical results are in Supplementary Table S7 and the main text.

We measured the survival of (*simw^501^*); *OreR* adults and genotypic controls after infection with the natural *Drosophila* bacterial pathogen *P. rettgeri*, as well as adult flies that were given a sham infection. The majority of deaths occurred 3–4 days post infection, consistent with prior studies using this pathogen (Duneau et al. 2017a). The proportion of flies surviving infection was significantly affected by mito–nuclear genotype (mtDNA* *×* *nuclear* *×* *infection, *P *=* *0.014), with greater mortality in the energetically-compromised genotype (Fig. 5 and Supplementary Tables S7 and S8). While the four-way interaction with sex was not significant, the magnitude of the effect of infection on (*simw^501^*); *OreR* females was larger than it was in males (Supplementary Tables S7 and S8). Survival analyses also indicated that the hazard ratio associated with infection was larger for individuals with the energetically-compromised genotype, relative to other genotypes, and larger for females of this genotype, relative to males (female hazard ratio* *=* *4.72, male hazard ratio* *=* *3.76) (Supplementary Fig. S4 and Table S9).

### Compromised energy metabolism revealed an immunity–fecundity tradeoff

**Fig. 6:**
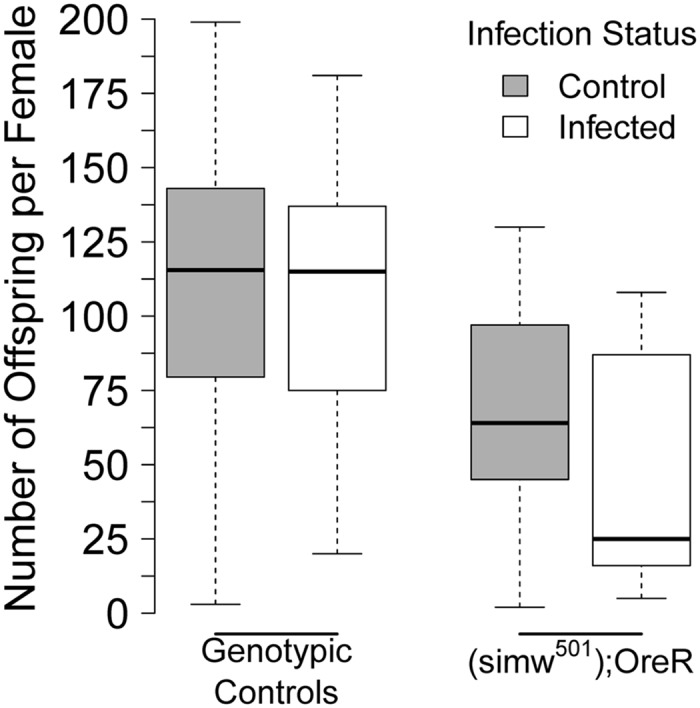
Compromised energy metabolism in (*simw^501^*); *OreR* revealed an immunity–fecundity tradeoff. Surviving infection decreased the total number of offspring produced by (*simw^501^*); *OreR* females, relative to sham-infected females, an effect that was not observed in control genotypes with normal metabolism. Data from 15–20 replicate females for each genotype across 3 days of egg laying are presented in Supplementary Fig. S4. Statistical results are in Supplementary Table S10 and in the main text.

We measured the offspring produced by females that survived for 5* *days following bacterial or sham infection. There was a significant interaction effect between mtDNA, nuclear genotype, and infection treatment on the number of offspring produced by females (mtDNA* *×* *nuclear* *×* *infection, *P *=* *0.0056). This interaction was only significant when (*simw^501^*); *OreR* females were included in the analysis (Supplementary Table S10). In control genotypes, there was no evidence for a tradeoff between immunity and fecundity; over the course of 3* *days, females with control genotypes produced similar numbers of offspring whether they had survived a sham infection or a pathogen infection (infection, *P *=* *0.99), a pattern that was independent of mito–nuclear genotype (mtDNA* *×* *nuclear* *×* *infection, *P *=* *0.10) (Fig. 6 and Supplementary Fig. S5 and Table S9). However, (*simw^501^*); *OreR* females that survived infection with *P. rettgeri* had fewer offspring than sham-infected females of the same genotype (Fig. 6) (infection, *P = *0.049), an effect that was larger on the second and third days of egg production (Fig. 6 and Supplementary Fig. S5 and Table S10).

## Discussion

Life-history tradeoffs occur due to differential resource allocation to the competing demands of organismal growth, maintenance, performance, and reproduction (Harshman and Zera 2007; King et al. 2011). These tradeoffs can vary among genotypes or within an individual across life stages (Zera and Larsen 2001), and can be modified by environmental stressors, such as temperature (Partridge et al. 1995; Adamo and Lovett 2011), pathogens (Love et al. 2008; McKean et al. 2008; Valtonen and Rantala 2012; Schwenke et al. 2016), and decreased resource availability (Burger et al. 2007). The latter can have particularly strong effects on reproductive fitness that can range from gonadal development (Bergland et al. 2008) to the production of sexual ornaments and signals (Siva-Jothy 2000; Fedorka and Mousseau 2007; Emlen et al. 2012; Gilbert and Uetz 2016; Gilbert et al. 2016). Decreased dietary resources negatively impact ovary development and the number of eggs produced by female *Drosophila* (Drummond-Barbosa and Spradling 2001; Bergland et al. 2008). In other insects, decreased access to nutritional resources can lower immune activation (Jacot et al. 2005), change gene expression related to immune function (Adamo et al. 2016), and reveal costs of immunity (Moret and Schmid-Hempel 2000). However, immunity–fecundity tradeoffs in insects can also be independent of resource availability (Stahlschmidt et al. 2013). Finally, some insect larvae have diet preferences that maximize the appropriate immune response (Cotter et al. 2011). These observations indicate that energetic–immune interactions are likely important in shaping evolutionary responses to environmental challenges, as well as mediating life-history tradeoffs.

However, nutrient reduction is not always detrimental to immunity (Adamo et al. 2016) or fecundity (May et al. 2015). Short-term starvation can increase survival of infection (Brown et al. 2009), and decreased nutrition can increase generalized immune responses, such as phenyloxidase production (Miller and Cotter 2017a) and encapsulation (Saastamoinen and Rantala 2013), despite the fact that immune responses are energetically expensive (Cutrera et al. 2010; Kvidera et al. 2017). Decreased host cellular resources may also impact pathogen growth independent of changes in host immune function. It is possible that differences between studies are due to differences in the type (generalized vs. specific) of immune response under investigation (Lee 2006), but could also be due to other life-history differences between species (Hawley and Altizer 2011), as well as differences in constitutive versus induced immunity. Our results indicate that genetic variation in mitochondrial and nuclear genomes impacts survival of infection with a natural bacterial pathogen and reveals immunity–fecundity tradeoffs in female *Drosophila*, likely due to a compromised mitochondrial ability to convert environmental nutrients to cellular resources. While the genotypes in our experiments enable us to infer that the observed effects are due to disrupted mitochondrial protein synthesis, future experiments with additional energetic mutants will be important to test the generality of our findings.

In response to the natural bacterial pathogen *P. rettgeri*, *Drosophila* activate the Toll, IMD, and JAK/STAT pathways in the first day of infection and the degree of activation is predictive of survivorship (Sackton et al. 2010; Duneau et al. 2017a). However, natural populations harbor significant genetic variation for surviving infection by *P. rettgeri* and these genetic effects are modified by diet (Howick and Lazzaro 2014). Our results suggest that mutations that impact mitochondrial function may be an important source of genetic variation for immune function in natural populations. Mitochondria have been linked to innate and adaptive immune responses (West et al. 2011; Pourcelot and Arnoult 2014; Weinberg et al. 2015), although mitochondrial genotype does not always affect post-infection reproduction (Nystrand et al. 2017). While we infer that reduced survival and fecundity of infected (*simw^501^*); *OreR* females is due to a compromised energy supply that cannot meet the competing demands of immune function and reproduction, we did not directly measure immune responses in this study. Mitochondria have other roles that may contribute to our observations, including reactive oxygen species production, mitochondrial antiviral signaling, and cellular damage responses (West et al. 2011; Pourcelot and Arnoult 2014; Weinberg et al. 2015). Furthermore, changes in host cellular energetics may have effects on pathogen growth that are independent of host immune function.

Our results suggest that TOR signaling may be less responsive in energetically inefficient genotypes. External and internal energy sensing is integrated by TOR (Xu et al. 2012; Rider 2016) to regulate growth (Zhang et al. 2000; Kavitha et al. 2014), fecundity (Zhai et al. 2015), and autophagy (Neufeld 2010), and there is some indication of a role for TOR signaling in immunity (Cobbold 2013; Allen et al. 2016). TOR signaling is sensitive to many factors, including decreased nutrition (Nagarajan and Grewal 2014), mitochondrial dysfunction (Kemppainen et al. 2016), and overnutrition (Jia et al. 2014), and populations of *D.melanogaster* harbor genetic variation, including mitochondrial, that influences energy sensing via TOR (Villa-Cuesta et al. 2014b; Stanley et al. 2017). Thus, TOR signaling is an important pathway integrating external and internal energetic and immunity status that may influence the evolution of life-history traits in response to the environment. Our results are consistent with other studies that indicate that this pathway may be limited in the extent to which the addition of multiple inputs can continue to cause increased signaling via TOR. Both simulated low nutrition via rapamycin (Villa-Cuesta et al. 2014a) and genetic manipulation of TOR (Nagarajan and Grewal 2014) fail to generate the expected phenotypic effects of nutrient limitation. Together, these observations indicate that there may be a threshold for nutrient sensing that, once crossed, prevents further repression of TOR. An alternative hypothesis is that mitochondrial protein synthesis, which is the target of this genetic incompatibility, may act downstream of TOR signaling; in *Drosophila*, cytoplasmic tRNA synthesis and subsequent protein synthesis are downstream of TOR and are necessary for nutrient-dependent growth regulation via this nutrient-sensing pathway (Rideout et al. 2012).

In our study, infection reduced (*simw^501^*); *OreR* survival more strongly in females than in males. In general, male *Drosophila* survive infection better than do females (Short and Lazzaro 2010; Vincent and Sharp 2014; Duneau et al. 2017b), a pattern that we also observed. The higher survival of males could result from sex-specific differences in immune expression due to Y-linked regulation (Fedorka and Kutch 2015), differences in antimicrobial peptide production (Jacobs et al. 2016; Duneau et al. 2017b), or potentially from differential suppression of the immune system by juvenile hormone, which has been shown to underlie differences in immune function between mated and un-mated females (Schwenke and Lazzaro 2017). An energetic explanation may be that females have less excess supply to invest in immune function, due to differential costs of gamete production (Bateman 1948; Rolff 2002; McKean et al. 2008; Hayward and Gillooly 2011; Schwenke et al. 2016). Consistent with this idea, mated females have lower antimicrobial peptide production than non-mated females (Short and Lazzaro 2010), and our prior results indicate that compromising cellular energy metabolism has greater effects on female reproduction, relative to male reproduction (Hoekstra et al. 2018).

These patterns are counter to the expectation that female *Drosophila* might mount stronger immune responses, because the resulting increase in longevity would provide greater lifetime opportunity for reproduction (McKean and Nunney 2005), a pattern that has been observed in many species (Klein 2004; Nunn et al. 2009; Miller and Cotter 2017b). In fact, investment in immunity has been shown to be greater in the sex that has higher investment in offspring, regardless of sex (Roth et al. 2011). However, this pattern may not be observed across all conditions, as environmental effects, such as stress, can decrease immune responses (Husak et al. 2017). Furthermore, in a study where female *Drosophila* appeared to invest more in immune function than did males, the effects were influenced by the presence of *Wolbachia* (Gupta et al. 2017). While none of our genotypes are infected with *Wolbachia*, understanding the interactions between this endosymbiont and mitochondrial effects on host energetics, immunity, and reproduction would provide important insight on the spread of *Wolbachia* in natural populations. An energetic framework that considers how external environmental conditions and internal conditions, such as sex, endosymbiont status, and tissue (e.g., ovary vs. testes) affect the balance of energy supply and demand (Hoekstra et al. 2018), may be a powerful framework for predicting under what conditions sexes may differ in their immune investment and when genetic variation in mitochondrial function will have sex-specific effects on immune function and tradeoffs between reproduction and immunity (Cressler et al. 2014; Tate and Graham 2015).

## Author contributions

J.L.B., C.D.M., and K.L.M. conceived and designed the study and analyzed the data. J.L.B. and C.D.M. carried out the experiments. J.L.B. and K.L.M. drafted the initial version of the manuscript, and all authors revised and gave the final approval for publication.

## Supplementary Material

Supplementary Data

## Supplementary data

Data available from the Dryad Digital Repository at https://datadryad.org/resource/doi:10.5061/dryad.88mk4dh.
